# Flaxseed oil and α-lipoic acid combination reduces atherosclerosis risk factors in rats fed a high-fat diet

**DOI:** 10.1186/1476-511X-11-148

**Published:** 2012-10-31

**Authors:** Jiqu Xu, Wei Yang, Qianchun Deng, Qingde Huang, Jin’e Yang, Fenghong Huang

**Affiliations:** 1 Department of Product Processing and Nutriology, Oil Crops Research Institute, Chinese Academy of Agricultural Sciences, 2 Xudong Second Road, Wuhan, 430062, P.R. China; 2 Hubei Key Laboratory of Lipid Chemistry and Nutrition, Oil Crop s Research Institute, Chinese Academy of Agricultural Sciences, 2 Xudong Second Road, Wuhan, 430062, P.R. China; 3 Department of Nutrition and Food Hygiene, School of Public Health, Tongji Medical College, Huazhong University of Science and Technology, 13 Hangkong Road, Wuhan, 430030, P.R. China

**Keywords:** Flaxseed oil, α-lipoic acid, Atherosclerosis, Oxidant stress, Plasma lipids, Inflammation

## Abstract

**Background:**

Atherosclerosis is a major manifestation of the pathophysiology underlying cardiovascular disease. Flaxseed oil (FO) and α-lipoic acid (LA) have been reported to exert potential benefit to cardiovascular system. This study tried to assess the effect of supplement of FO and LA combination on the atherosclerosis risk factors in rats fed a high-fat diet.

**Methods:**

LA was dissolved in flaxseed oil to a final concentration of 8 g/kg (FO+LA) when used. The rodent diet contained 20% fat. One-fifth of the fat was soybean oil and the others were lard (HFD group), or 75% lard and 25% FO+LA (L-FO+LA group), or 50% lard and 50% FO+LA (M-FO+LA group), or FO+LA (H-FO+LA group). Animals were fed for 10 weeks and then killed for blood collection.

**Results:**

Supplement of FO and LA combination significantly enhanced plasma antioxidant defense capacities, as evaluated by the marked increase in the activities of SOD, CAT and GPx as well as the level of GSH, and the significant reduction in lipid peroxidation. Simultaneous intake of FO and LA also reduced plasma TG, TC and LDL-C contents and elevated the ratio of HDL-C/LDL-C. Besides, in parallel with the increase of FO and LA combination, plasma IL-6 and CRP levels were remarkably reduced.

**Conclusion:**

Supplement of FO and LA combination may contribute to prevent atherogenesis by improving plasma oxidative stress, lipid profile and inflammation.

## Introduction

Cardiovascular disease (CVD) is widely recognized as the leading cause of premature death and a major cause of disability in most developed and developing countries. Atherosclerosis, a manifestation of the pathophysiology underlying CVD, constitutes the single most important contributor to this growing burden of this disease. Recent studies suggest that oxidant stress [1], lipid abnormalities [2] and chronic inflammation [3] play important roles in the etiology of atherosclerosis and subsequent CVD.

Flaxseed oil (FO) is the main component of the flaxseed and one of the world's most important vegetable sources of α-linolenic acid (LNA, 18:3n-3). As a nutritionally essential polyunsaturated fatty acid (PUFA), LNA can act as the precursor of longer chain n-3 PUFA (EPA and DHA) or compete with linoleic acid or direct interaction with ion channels and nuclear receptors, and thus may exert various biological functions in the human body, such as accelerating brain growth in preterm and neonatesand, antiarrhythmic functions and neuroprotective functions [4]. In addition, LNA is also reported to have beneficial effects on blood lipid profiles [4-7] and inflammation [4,8,9], which may responsible for the protection against CVD bestowed by FO. However, on the other hand, since LNA is highly susceptible to oxidation, FO addition leads to a significantly higher tendency toward plasma lipid peroxidation [10,11], which may have an adverse effect on the protection of cardiovascular system.

α-lipoic acid (LA), also referred to as thioctic acid, is a disulfide compound that is found naturally in mitochondria as the coenzyme for pyruvate dehydrogenase and α-ketoglutarate thus serves a critical role in mitochondrial energy metabolism. Although orally supplied LA may not be used as a metabolic cofactor, there are a unique set of biochemical activities with potential pharmacotherapeutic value against a host of pathophysiologic insults [12]. For example, LA has gained considerable attention as an excellent antioxidant to reduce oxidative stress [13-15]. Further, LA is fat- and water-soluble, which makes it effective against a broader range of free radicals. Early studies have shown the capacity of LA to decrease plasma lipids in rats [16-18]. Besides, LA also exhibits an anti-inflammatory activity in clinical trial [19]. These mechanisms make LA possess the potential abilities for antiatherogenesis.

To our knowledge, the effects of a simultaneous intake of FO and LA on cardiovascular system have not been investigated. Therefore, the objectives of this study were to determine that whether FO and LA combination can reduce atherosclerosis risk factors in rats fed a high-fat diet.

## Materials and methods

### Chemical sources

Commercial deodorized lard was purchased from a local supermarket. The flaxseed oil was obtained from Caoyuankangshen Food Co., Ltd., and its fatty acid compositions were listed in Table 1. LA was purchased from Sigma-Aldrich (St. Louis, MO, USA) and was dissolved in flaxseed oil to a final concentration of 8 g/kg (FO+LA) when used.

**Table 1:** Fatty acid compositions of flaxseed oil

**Fatty acid**	**Composition (wt.%)**
Palmitic acid (C16:0)	6.1
Stearic acid (C18:0)	3.7
Oleic acid (C18:1)	22.4
Linoleic acid (C18:2)	14.9
Linolenic acid (C18:3)	52.9

### Animals and diets

The experiment was conducted with 40 male Sprague–Dawley rats (initially weighing 150–170 g). The animals were housed individually and maintained at a controlled ambient temperature (24 ± 1 °C) under diurnal conditions (light–dark: 08:00–20:00) with access to laboratory chow and tap water ad libitum. After 1 week of acclimatization, rats were randomly divided into a high-fat diet (HFD) group and three experimental groups (n =10 per group). All animals were fed purified experimental diets which contained 35% maize starch, 20% casein, 15% sucrose, 5% cellulose, 3.5% mineral mixture (AIN-93M), 1% vitamin mixture (AIN-93M), 0.3% DL-methionine, 0.2% choline bitartrate and 20% fat. One-fifth of the fat in the diet of each group was soybean oil and the others were lard (HFD group), or 75% lard and 25% FO+LA (L-FO+LA group), or 50% lard and 50% FO+LA (M-FO+LA group), or FO+LA (H-FO+LA group). Every week, all ingredients for the purified diets were mixed, formed into a dough with purified water, rolled into pellets, sealed in air-tight plastic bags under nitrogen gas and stored at −80°C until use. The food in the animal cages was shaded from light and changed every day. The animals were cared for in accordance with *the Guiding Principles in the Care and Use of Animals*. The experiment was approved by the local animal care committee.

### Blood processing

After 10 weeks of feeding, animals, fasted for 16 hours, were killed under anaesthesia, blood was collected in the presence of sodium heparin from the heart immediately. Blood samples were centrifuged at 1500 g for 10 min at 4°C and the plasma was stored at −80°C until analysis.

### Plasma lipids analysis

The plasma triglyeride (TG), total cholesterol (TC), low-density lipoprotein cholesterol (LDL-C) and high-density lipoprotein cholesterol (HDL-C) levels were measured using commercial enzymatic kits (Zhongsheng Beikong Biotech Company, China).

### Assay of liver antioxidant capacity and lipid peroxidation

Superoxide dismutases (SOD) activity was measured according to the method of Kono [20]. Catalase (CAT) activity was estimated basing on the method of Goth [21]. Glutathione peroxidase (GPx) activity was measured by the method of Sazuka [22]. The glutathione (GSH) content was determined by the method of Moron [23]. The total antioxidant capability (T-AOC) was assayed with commercial kits (Nanjing Jiancheng Bioengineering Institute, China).Thiobarbituric acid reactive substances (TBARS) level was estimated by the method of Buege [24]. The detection procedure of these enzymes activities has been described in detail in our previous report [25].

### Assay of protein concentration

The protein concentration was determined by the method of Lowry [26] with bovine serum albumin as the standard.

### Assay of plasma inflammatory markers

The C-reactive protein (CRP) and interleukin 6 (IL-6) levels in plasma were measured with the use of commercially available rat CRP ELISA kit (eBioscience San Diego, CA) and rat IL-6 ELISA kit (eBioscience San Diego, CA), respectively. All the procedures and conditions were consistent with the instructions of these kits.

### Statistical analyses

Values are presented as mean ± SEM (standard error of the mean). The data were analyzed by one-way ANOVA, followed by the Fisher PLSD post hoc test if the overall differences were significant (*p*< 0.05). All statistical analyses were performed using SPSS 13.0 statistical software (SPSS Inc., Chicago, IL) and a difference was considered significant when *p* < 0.05.

## Results

### Plasma antioxidative capacity and lipid peroxidation

**Figure 1:**
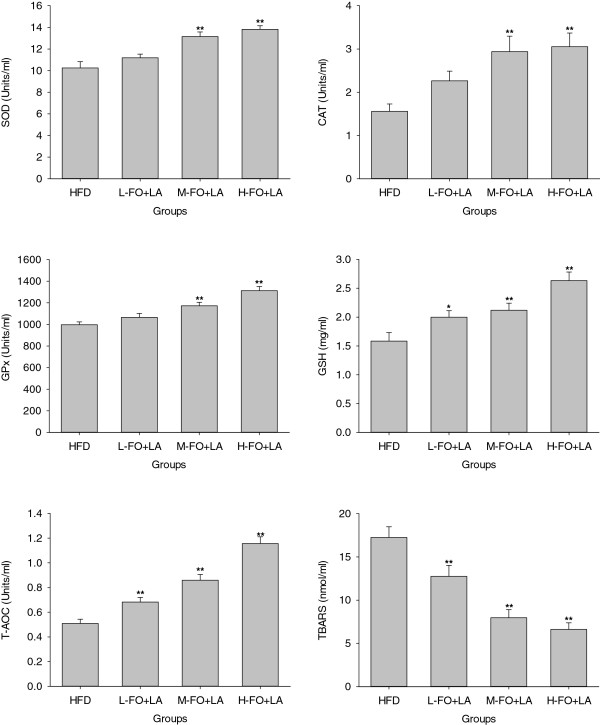
**Effects of FO and LA combination on the activities of antioxidant enzymes (SOD, CAT and GPx), the levels of GSH, T-AOC and contents of TBARS in plasma of rats fed a high-fat diet.** HFD: high-fat diet group; L-. M- and H- FO + LA: low, middle and high contents of FO and LA combination groups. Bars represent the mean ± SD from 10 animals in each group. * *p* < 0.05 and ** *p* < 0.01 compared to the HFD group

As shown in Figure 1, animals in M- and H- FO+LA groups revealed significantly higher plasma antioxidant enzymes SOD, CAT and GPx activities than their counterparts in HFD group. Administration of L-, M- and H- FO+LA for 10 weeks significantly augmented the GSH levels in plasma when compared to rats treated with HFD. As the overall antioxidative capacity, plasma T-AOC in all three FO+LA groups was markedly higher than that in HFD group. Moreover, when plasma TBARS were evaluated as the marker of lipid peroxidation, there were significant decline in the levels of plasma TBARS in all three FO+LA groups when compared with those in HFD group.

### Plasma lipids

**Figure 2:**
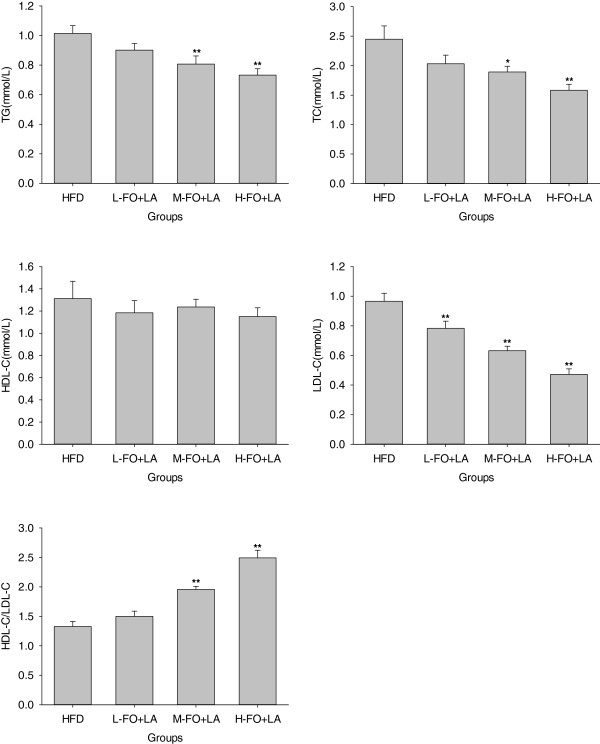
**Effects of FO and LA combination on various plasma lipid parameters (TG, TC, LDL-C and HDL-C) and on the ratios of plasma HDL-C/LDL-C of rats fed a high-fat diet.** HFD: high-fat diet group; L-. M- and H- FO + LA: low, middle and high contents of FO and LA combination groups. Bars represent the mean ± SD from 10 animals in each group. * *p* < 0.05 and ** *p* < 0.01 compared to the HFD group

The effects of FO+LA on the levels of plasma lipids are shown in Figure 2. Animals in M- and H-FO+LA groups exhibited significantly lower plasma TG and TC levels than that in HFD group. There were no marked differences in the level of plasma HDL-C in all groups, while rats in all FO+LA groups showed significantly lower LDL-C levels than that in HFD group. As results, M- and H-FO+LA groups had significantly higher ratios of HDL to LDL cholesterol than HFD group.

### Plasma inflammatory

**Figure 3:**
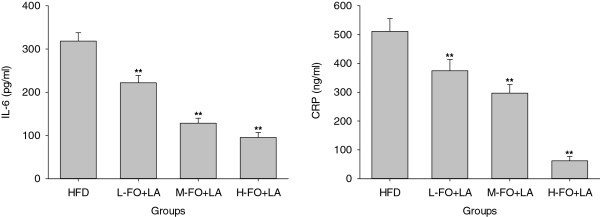
**Effects of FO and LA combination on the level of IL-6 and CRP in plasma of rats fed a high-fat diet.** HFD: high-fat diet group; L-. M- and H- FO + LA: low, middle and high contents of FO and LA combination groups. Bars represent the mean ± SD from 10 animals in each group. ** *p* < 0.01 compared to the HFD group

As can be seen in Figure 3, there were significantly decline in the plasma levels of IL-6 and CRP in all three FO+LA groups when compared with those in HFD group.

## Discussion

FO has gained more and more attention in many areas recently primarily because it is one of the richest sources of LNA. As the plant precursor of EPA and DHA, ALA may produce significant cardiovascular benefits which have been proved by some clinical trials, epidemiological investigations and experimental studies [27]. LA is a potent natural antioxidant and represents a possible protective agent against risk factors of CVD [28]. In this report, we examined the effect of FO and LA combination on atherosclerosis risk factors in rats fed a high-fat diet.

The imbalance between cellular production of free radicals and the antioxidant defense is referred to as oxidative stress. The relative excessive production of free radicals can attack all types of biomolecules, which may affect oxidation of low density lipoproteins and lead to the destruction of cellular components and thus initiates the processes of atherogenesis. Growing evidence indicates that oxidative stress is the pivotal pathogenetic factor and unifying mechanism for atherosclerosis and other cardiovascular diseases [29]. For example, free radicals are key mediators of various signaling pathways which underlie vascular inflammation in atherogenesis [30]. However, an efficient antioxidant defense mechanism (enzymatic and non-enzymatic) is able to counteract the deleterious effects of oxidative stress. The primary antioxidant enzymes in mammals include SOD which converts superoxide to hydrogen peroxide, GPx and CAT which are responsible for converting hydrogen peroxide to water [31]. GSH is a very important non-enzymatic antioxidant which can react directly with free radicals or act as an electron donor in the reduction of peroxides catalyzed by GPx [32]. In the present study, FO and LA combination markedly increased the plasma activities of these antioxidant enzymes (SOD, CAT and GPx) as well as GSH level, which resulted in pronounced enhancement of plasma T-AOC. As a consequence, plasma lipid peroxidation levels significantly declined with the supplement of FO and LA combination. However, FO itself is hardly thought to have any antioxidative activity and further, it may cause lipid peroxidation because of its susceptibility to oxidation [10,11]. Therefore, LA should impart the entire antioxidative potency in this experiment. In fact, LA has been proved to be a potent multifunctional biological antioxidant. It can reduce oxidative stress by scavenging free radicals, chelating transition metals, increasing intracellular GSH and the redox regeneration of other antioxidants such as vitamins C and E [13-15,33,34]. In addition, LA also decreases oxidative stress by restoring enzymatic antioxidant system in blood in different status [34,35].

It is well known that hyperlipidemia is a highly predisposing condition for arteriosclerosis and other cardiovascular disease. A large body of evidence has been presented showing that high-fat diet rich in saturated fatty acid results in hyperlipidemia. In the present study, FO and LA combination decreased the plasma TG, TC and LDL-C levels, and both of them contributed to these beneficial changes. LNA and LA have both been reported to suppress the expression and activities of many hepatic fatty acid syntheses such as fatty acid synthase (FAS), malic enzyme and glucose 6-phosphate dehydrogenase [36-38], and hence decrease fatty acid synthesis in liver. On the other hand, LNA sharply enhances hepatic peroxisomal and mitochondrial fatty acid oxidation rate by increasing the expression and activities of a series of fatty acid oxidation enzymes [37,39], and LA also shows the similar action on inducing fatty acid oxidation [18]. In addition, FO has been shown to have hypocholesterolaemic effect, which might result from increases of hepatic LDL-receptor expression and cholesterol catabolism/output [7]. Administration of LA can likewise reduce the elevated plasma TC and LDL-C induced by high-fat diet by downregulating the expression of genes involved in cholesterol synthesis in liver [18]. Although simultaneous intake of FO and LA did not markedly affect plasma HDL-C level in the present study, the significant increase in the ratio of HDL to LDL cholesterol still meant that the combination of FO and LA had a marked protective effect with respect to atherosclerosis.

Based on strong experimental and clinical evidence, the general consensus is that inflammation plays a central role in both initiation and progression of the atherosclerotic process [40-42]. Various proinflammatory risk factors such as oxidized LDL and infectious agents can trigger the production of proinflammatory cytokines which are central mediators of inflammation associated with atherogenesis. As two of the most important proinflammatory cytokines, IL-6 and CRP are associated with CVD and served as inflammatory markers for prediction of atherosclerotic risk [43-45]. In the present study, supplement of FO and LA remarkably reduced the plasma levels of both IL-6 and CRP, which meant that combination of FO and LA has an ability to improve inflammation status. Supporting our results, intakes of dietary FO or LA have been shown to elicit antiinflammatory effects by inhibiting inflammatory cytokine production. For example, FO can suppress the expression of inflammatory cytokine such as IL-6, IL-1, CRP and TNF-α via a reduction in NF-κB induced gene expression and/or an activation of PPAR-γ [4,8,46]. Similarly, LA has also been demonstrated to inhibit NF-κB activation independent of its antioxidant function [47,48] and thus reduce circulating levels of inflammatory markers [19].

In summary, the combination of FO and LA is effective in amelioration of oxidative stress, lipid profile and inflammation of plasma in rats fed a high-fat diet. These results suggested that supplement of FO and LA combination might contribute to prevent atherogenesis and then decrease the incidence of CVD.

## Competing interest

No competing financial interests exist.

## Authors’ contributions

Author JX designed and wrote a first draft of the paper. WY, QD and JY carried out all the experiments. QH performed the data analysis and created the figures. FH contributed to the design of the study, reviewed the manuscript and contributed to the final version. All authors contributed to and have approved the final manuscript.
